# Tracing Five Decades of Psoriasis Pharmacotherapy: A Large-Scale Bibliometric Investigation with AI-Guided Terminology Normalization

**DOI:** 10.3390/ph18091422

**Published:** 2025-09-21

**Authors:** Ada Radu, Andrei-Flavius Radu, Gabriela S. Bungau, Delia Mirela Tit, Paul Andrei Negru

**Affiliations:** 1 Doctoral School of Biological and Biomedical Sciences, University of Oradea, 410087 Oradea, Romania; adaradu@uoradea.ro (A.R.); dtit@uoradea.ro (D.M.T.); negru.paulandrei@student.uoradea.ro (P.A.N.); 2 Department of Pharmacy, Faculty of Medicine and Pharmacy, University of Oradea, 410028 Oradea, Romania; 3 Department of Psycho-Neurosciences and Recovery, Faculty of Medicine and Pharmacy, University of Oradea, 410073 Oradea, Romania; 4 Department of Preclinical Disciplines, Faculty of Medicine and Pharmacy, University of Oradea, 410073 Oradea, Romania

**Keywords:** psoriasis, bibliometric analysis, psoriasis therapy, VOSviewer, Python, web of science

## Abstract

**Background/Objectives**: Large-scale bibliometric assessments of psoriasis pharmacotherapy research remain limited despite significant research output in this rapidly evolving field. This study aimed to map the evolution of systemic psoriasis therapy research over five decades and demonstrate how systematic analysis of research trajectories can illuminate the transformation of specialized medical fields into central components of precision medicine. **Methods**: A comprehensive bibliometric analysis was conducted using Web of Science Core Collection as the single data source, examining 19,284 publications spanning 1975–2025. The methodology employed AI-enhanced terminology normalization for standardizing pharmaceutical nomenclature, VOSviewer version 1.6.20 for network visualization, and Bibliometrix package for temporal trend analysis and thematic evolution mapping. International collaboration networks, thematic evolution across three distinct periods (1975–2000, 2001–2010, 2011–2025), and citation impact patterns were systematically analyzed. **Results**: Four distinct developmental phases were identified, with publications growing from 9 articles in 1975 to 1638 in 2024. The United States dominated research output with 5959 documents, while Canada achieved the highest citation efficiency at 62.65 citations per document. Global collaboration encompassed 70 countries organized into four regional clusters, with a 28-nation Asia–Pacific–Africa–Middle East alliance representing the largest collaborative group. Citation impact peaked during 2001–2008, coinciding with revolutionary biological therapy introduction. Thematic evolution demonstrated systematic transformation from two foundational themes to nine specialized domains, ultimately consolidating into four core areas focused on targeted therapeutics and evidence-based methodologies. Keyword analysis demonstrated progression from basic immunological studies to sophisticated targeted interventions, evolving from tumor necrosis factor alpha inhibitors to contemporary interleukin-17/interleukin-23 pathway targeting and Janus kinase inhibitors. **Conclusions**: Over five decades, psoriasis therapeutics research has shifted from a niche dermatological discipline to a central model for innovation in immune-mediated diseases. This evolution illustrates how bibliometric approaches can capture the dynamics of scientific transformation, offering strategic insights for guiding pharmaceutical innovation, shaping research priorities, and informing precision medicine strategies across inflammatory conditions.

## 1. Introduction

Psoriasis is a chronic autoimmune skin disorder marked by erythematous, scaly lesions due to accelerated epidermal turnover. It is non-infectious but often causes itching, pain, and psychosocial burden. The condition is immune-driven and strongly influenced by genetic factors [1,2]. Psoriasis ranks among the most common dermatological disorders, impacting a substantial portion of the global population [3]. Reported data show an increase from 92 to 99 new cases per 100,000 individuals between 1990 and 2017. Epidemiological projections suggest a potential rise in incidence rates by 2030 [4].

The disease is defined by various clinical forms, including inverse, erythrodermic, pustular, guttate, and plaque types. Several distinct types of psoriasis exist, each presenting unique characteristics. The most prevalent variant of psoriasis is plaque psoriasis [5].

Psoriasis has systemic implications that extend beyond the skin, involving multiple organ systems and mental health. Increasing evidence supports its classification as a systemic autoimmune disease driven by chronic inflammation. In addition to well-established associations with cardiometabolic, gastrointestinal, renal, oncological, and infectious conditions, recent findings also implicate the reproductive, oral, ocular, and even cutaneous systems. Psychiatric comorbidities are gaining recognition not only due to the emotional impact of skin lesions but also through shared immune-inflammatory mechanisms linking psoriasis and mental disorders [6]. Emerging evidence suggests a bidirectional link between psoriasis and psychiatric disorders, including depression, anxiety, and schizophrenia, yet prospective studies remain scarce. Inflammatory pathways (i.e., elevated cytokines, hypothalamic–pituitary–adrenal axis dysregulation, and shared immune-related genes) may underlie this association. Mental health comorbidities are prevalent yet often overlooked, despite their negative impact on treatment adherence and outcomes. Patients with psoriasis exhibit a 1.5-fold increased risk of depression, nearly double the likelihood of suicidal ideation, and anxiety disorders are present in up to 16% of cases [7].

Psoriasis arises from a complex interplay between genetic susceptibility and environmental triggers. Its pathogenesis involves dysregulated immune responses mediated by T cells and dendritic cells, along with contributions from galectin-3, vaspin, fractalkine, lipocalin-2, human neutrophil peptides, and antimicrobial peptides [8].

Psoriasis treatment includes a range of systemic, topical, and biologic options tailored to disease severity. Conventional oral agents such as methotrexate, cyclosporine, and acitretin act through immunosuppression or modulation of keratinocyte proliferation but are limited by organ toxicity, particularly hepatotoxicity and nephrotoxicity. Apremilast, a phosphodiesterase 4 inhibitor, reduces proinflammatory cytokines and has shown efficacy in psoriatic arthritis regardless of prior Disease-Modifying Antirheumatic Drug use. Topical therapies remain essential in mild-to-moderate cases. Vitamin D analogs like calcipotriol inhibit keratinocyte proliferation but may cause local irritation and variable response rates. Potent corticosteroids, although effective, are limited by risks such as skin atrophy and irritation. Tacrolimus, a calcineurin inhibitor, avoids these effects but has limited penetration in plaque-type lesions. Injectable immunosuppressants such as methotrexate and azathioprine offer rapid results but may induce severe reactions [4,9].

Biologics have transformed psoriasis care, offering improved disease management and quality of life for patients, with generally favorable safety profiles. However, challenges such as high cost, potential immunogenicity and complexity of administration have sparked interest in alternative treatment options. The fact that biologic agents interact with a specific cytokine, such as tumor necrosis factor-alpha (TNF-α), interleukin (IL)-17 or -23) in a targeted manner has revolutionized the capacity to treat psoriasis compared to the era of a more generalized immunosuppression reflected by the traditional oral medications. This represents an improved treatment regimen where targeted immunomodulation has resulted in a great enhancement in both safety and efficacy for biologic agents. Biologic agents approved for the treatment of psoriasis belong to four main pharmacologic classes: TNF-α inhibitors (i.e., adalimumab, etanercept, infliximab, certolizumab pegol), IL-17 inhibitors (i.e., secukinumab, brodalumab, and ixekizumab), IL-23 inhibitors (i.e., guselkumab, tildrakizumab, and risankizumab), and the IL-12/23 inhibitor ustekinumab [10].

Janus kinase (JAK) inhibitors represent another promising class of oral therapies targeting intracellular signaling pathways central to psoriatic inflammation. Tofacitinib, approved for psoriatic arthritis, inhibits JAK1/3 and has demonstrated efficacy in plaque psoriasis, though concerns regarding long-term safety have limited its approval for cutaneous forms. Upadacitinib and baricitinib have also shown benefit in psoriatic arthritis, while ruxolitinib targets T helper 17 cells-mediated inflammation, but broader JAK inhibition raises safety issues, including infection risk and hematologic abnormalities. Second-generation JAK inhibitors offer greater selectivity, potentially improving tolerability. Deucravacitinib, a U.S. Food and Drug Administration-approved tyrosine kinase 2 (TYK2) inhibitor, has shown robust efficacy in Phase III trials for moderate-to-severe plaque psoriasis, with a favorable safety profile. PF-06826647, another TYK2 inhibitor, achieved significant Psoriasis Area and Severity Index (PASI) reduction in Phase II trials. Brepocitinib, a dual TYK2/JAK1 inhibitor, demonstrated dose-dependent improvement with minimal adverse events. Among emerging oral molecules, piclidenoson (A3 adenosine receptor agonist), belumosudil (Rho-associated coiled-coil containing protein kinase 2 inhibitor), and cedirogant (retinoic acid receptor-related orphan receptor gamma t inverse agonist) show immunomodulatory potential in early trials. These agents aim to fine-tune inflammatory responses while minimizing systemic toxicity. Continued development of targeted oral therapies may broaden the therapeutic landscape for patients with moderate to severe psoriasis inadequately controlled by conventional agents [11].

Despite significant therapeutic advances, psoriasis remains an incurable, chronic inflammatory condition requiring long-term management and continuous innovation in treatment approaches. Pharmacotherapy is rapidly evolving, with emerging strategies such as drug repurposing, nanotechnology-based delivery systems [12], and personalized medicine offering new avenues for disease control [13]. In this dynamic landscape, it is essential to systematically evaluate the growing volume of scientific output to ensure timely integration of new insights into clinical practice. Bibliometric analyses serve as valuable tools to monitor research trends, quantify scientific productivity, and assess the impact and collaboration patterns within the field [14], mapping the evolution of treatment strategies.

Bibliometric analysis has emerged as an increasingly important approach for mapping research landscapes in psoriasis. However, existing bibliometric investigations in psoriasis demonstrate significant methodological and scope limitations that constrain comprehensive understanding of the field’s evolution. The majority of studies focus on narrow research subdomains, including systematic review quality assessment [15,16], specific therapeutic classes such as biologics [17,18], or specialized topics like metabolomics [19] and nail psoriasis [20]. Temporal scope represents a critical limitation, with most investigations covering periods of 10–20 years, insufficient for capturing the complete transformation from conventional systemic therapies through biological therapy emergence to contemporary precision targeting approaches. For instance, recent studies by Huang et al. [21] and Li et al. [22] examine only 2004–2023 and 2003–2022 respectively, thus not focusing on foundational research periods essential for understanding therapeutic evolution. Notably, no known previous investigation has employed AI-enhanced terminology normalization techniques essential for managing the complex and evolving pharmaceutical nomenclature characteristic of psoriasis therapeutics research. Geographic representation also remains limited, with studies focusing on specific regions, with Daou et al [23]. examining Arab countries and Ram [24] analyzing Indian contributions. More specifically, large-scale bibliometric investigations specifically focused on the pharmacological treatment of psoriasis remain notably limited, either due to their narrow focus on specific subdomains (i.e., metabolomics in psoriasis) [19], restricted temporal scope [25], or different methodological frameworks that do not directly center on psoriasis pharmacotherapy [26]. Given the ongoing expansion of the literature and the translational relevance of synthesizing current evidence, there remains an unmet need for a comprehensive mapping of the thematic evolution within this field.

A high-resolution, large-scale bibliometric and network analysis of global research on systemic pharmacotherapy for psoriasis, covering five decades of scientific output, was conducted. The objective was to trace structural shifts in research focus, identify emerging thematic clusters, and assess key bibliometric indicators such as publication trends, citation metrics, and international collaboration networks. By leveraging an extensive dataset, this work addresses the limited bibliometric representation of psoriasis pharmacotherapy. In addition, the use of AI-enhanced terminology standardization ensures high fidelity in thematic mapping, enabling precise tracking of conceptual transitions.

## 2. Results

### 2.1. Temporal Publication Analysis

**Figure 1:**
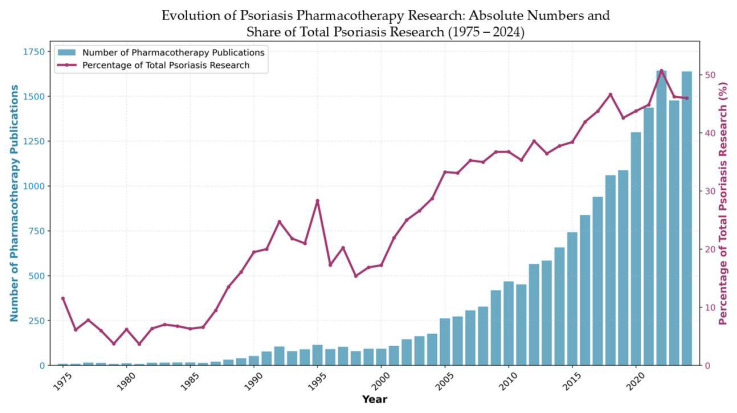
Temporal evolution of scientific publications (number) in systemic psoriasis therapy research (1975–2024).

This bibliometric analysis encompasses 19,284 publications spanning five decades (1975–2025), demonstrating the substantial evolution of scientific interest in systemic psoriasis therapies from a specialized dermatological concern to a major focus of contemporary immunological research. The temporal distribution (Figure 1) reveals four distinct developmental phases, with annual publication volumes growing from 9 articles in 1975 to a peak of 1638 in 2024; this increase reflects the field’s transformation.

The most dramatic acceleration occurred during 2005–2015, when publications nearly tripled, coinciding directly with the clinical introduction of revolutionary biologics including etanercept, infliximab, and adalimumab, which transformed treatment paradigms from conventional immunosuppression to targeted cytokine inhibition. This period established the foundation for contemporary psoriasis research, followed by continued momentum through 2016–2024, where output expanded from 837 to 1638 publications, reflecting the rapid diversification of therapeutic targets including IL-17, IL-23 pathway inhibitors, and JAK inhibitors.

The sustained research intensity, with 2024 representing the most productive year in the field’s history, indicates that systemic psoriasis therapy research has achieved mature scientific status while maintaining dynamic growth. Importantly, proportional analysis reveals that pharmacotherapy research has increased its share of total psoriasis research from 1975 to 2024, representing a 4-fold increase in relative research attention and peaking at 50.7% in 2022. This demonstrates that the observed exponential growth reflects genuine field prioritization toward therapeutic interventions rather than simply mirroring general expansion in psoriasis research, with modern pharmacotherapy now representing the dominant focus within the broader psoriasis research landscape. This exponential trajectory corresponds to major therapeutic breakthroughs, regulatory milestones, and the evolving understanding of psoriasis as a systemic immune-mediated disease requiring sophisticated treatment approaches beyond traditional dermatological interventions.

**Figure 2:**
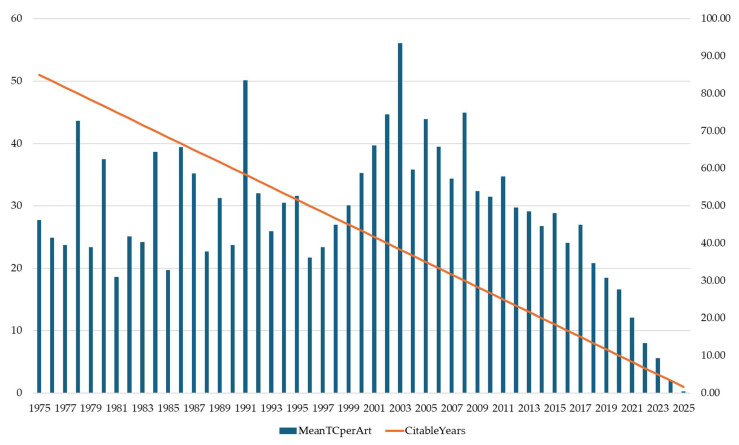
Citation impact evolution in systemic psoriasis therapy research, mean citations per article (1975–2025).

The temporal evolution of citation patterns (Figure 2) reveals the progressive scientific maturation and influence of systemic psoriasis therapy research across five decades. Mean Total Citations per Article (MeanTCperArt) demonstrates distinct phases of research impact, beginning with variable early influence (31.00–83.52 citations) during the foundational period (1975–1990), progressing through a peak impact era (1991–2008) with citation rates reaching 93.47 per article in 2003, and transitioning to contemporary research that shows expected citation lag effects in recent publications.

The field achieved its highest scientific influence during 2001–2008, with citation rates consistently exceeding 65 citations per article and peaking at 93.47 in 2003, coinciding with the introduction of revolutionary biological therapies and fundamental advances in psoriasis immunopathogenesis understanding. This period of exceptional impact reflects the transformative nature of research published during the early biological therapy era, when landmark studies establishing TNF-α inhibitors and other targeted treatments achieved widespread clinical adoption and academic recognition. Notable secondary peaks occurred in 1991 (83.52 citations) and 2008 (74.96 citations), indicating sustained high-impact research throughout the biological therapy development phase.

The declining citation rates observed from 2015 onward (48.03 to 0.50 by 2025) primarily reflect the natural citation accumulation lag rather than diminished research quality, as recent publications require several years to achieve full citation potential. The substantial citation impact achieved during the peak period demonstrates that systemic psoriasis therapy research successfully transitioned from a specialized dermatological field to a central component of immunological and pharmaceutical science, with foundational studies continuing to influence contemporary clinical practice and research directions.

### 2.2. Global Research Landscape, Productivity and Scientific Impact

An analysis of the geographic landscape (Table 1) of systemic psoriasis therapy research reveals that the United States is the dominant contributor in terms of publication volume. With 5959 documents, the U.S. has produced more than double the output of the next leading country and demonstrates a powerful research ecosystem, evidenced by its field-leading total citations (300339) and the most extensive international collaboration network (TLS of 6455). This indicates a well-funded and highly interconnected research infrastructure. The nation’s research also maintains a high impact, achieving an average of 50.40 citations per document.

While the U.S. leads in sheer quantity, several other nations distinguish themselves through exceptional research impact. Canada stands out for its remarkable citation efficiency, recording the highest average citation rate (62.65 citations per document) among all leading countries, signifying that its research has a disproportionately high influence.

A strong cohort of European nations forms the core of global research in this area. Germany and England present well-rounded profiles, combining high productivity (1819 and 2021 publications, respectively) with significant citation impact (54.08 and 51.23) and robust collaborative networks. In contrast, China’s rapid research expansion coincides with growing domestic pharmaceutical development in biologics and biosimilars. Despite ranking fifth in publication volume with 1598 documents, it has limited international collaboration score. This pattern suggests a high volume of research that has not yet achieved significant global traction or influence.

**Figure 3:**
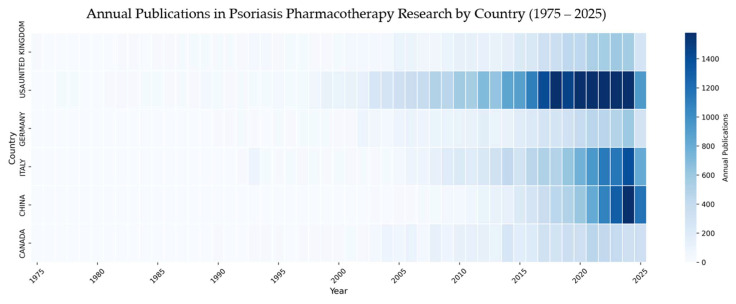
Heat map illustrating global research leadership trends in the pharmacological management of psoriasis (1975–2025). Each cell represents the number of new publications per country per year, with color intensity (Blues colormap) indicating publication volume. *X*-axis shows years at 5-year intervals, *Y*-axis lists countries. Generated using Python 3.12.3 with Seaborn and Matplotlib libraries.

The temporal evolution of research output in psoriasis pharmacotherapy from 1975 to 2025 is visualized in Figure 3. The analysis reveals distinct phases of growth and a significant shift in the geographic distribution of research activity over time. During the foundational period (1975–2000), the field was predominantly led by the United States and the United Kingdom. The USA established an early lead, accumulating 655 publications by 2000, while the UK followed with a cumulative output of 339 publications. European counterparts like Germany and Italy entered the field more gradually, with Germany’s output becoming notable in the 1990s and Italy showing a consistent presence.

A dramatic transformation of the research landscape is evident after 2010, characterized by an exponential increase in publication velocity, particularly from China. After having a marginal presence for decades (14 cumulative publications by 2000), China’s research output experienced a dramatic surge. The nation’s cumulative publications grew from 822 in 2015 to 9043 by 2025, marking a more than 10-fold increase in a single decade. This explosive growth indicates a massive strategic investment in dermatological and pharmacological research. Concurrently, Italy also demonstrated substantial acceleration, with its annual publication rate surpassing that of both Germany and the UK in the final decade. For instance, in 2024 alone, Italy produced 1386 articles, substantially more than the UK (562) and Germany (597). Throughout this period, the USA maintained its position as the overall volume leader, but the field evolved from a bipolar, Western led domain into a multipolar landscape with the recent and rapid emergence of powerful new research hubs, complemented by sustained growth from established contributors like Canada, which expanded its output significantly between 2000 and 2025.

### 2.3. Scientific Impact Analysis

Among the top publication sources for systemic psoriasis therapies, The Journal of the American Academy of Dermatology (JAAD) stands out as the most influential, combining both volume and impact. With 540 publications and 41,319 citations since 1980, JAAD leads in h-index (105), g-index (173), and maintains the highest m-index (2.283), indicating a consistently high citation rate relative to its age. The British Journal of Dermatology (BJD), despite producing the largest number of articles (645), has a slightly lower citation total (35,348) and an h-index of 93, suggesting a broader but somewhat less concentrated impact. Similarly, The Annals of the Rheumatic Diseases (ARD) demonstrates strong citation efficiency, with 266 articles generating 28,451 citations (averaging 106.9 citations per document), and ranks second in h-index (95), reflecting its pivotal role in immunology-oriented psoriasis research.

Several specialized journals show notable performance trends. The Journal of the European Academy of Dermatology and Venereology (JEADV) has rapidly gained influence since 1997. Rheumatology-focused outlets, such as The Journal of Rheumatology and Rheumatology, show robust performance. High-impact generalist journals like The Lancet, though contributing fewer articles (53), achieve the highest citation-per-document ratio (382.1), underscoring the visibility and influence of landmark studies published in broader clinical platforms. This distribution reflects a dual publication dynamic: dermatology and rheumatology journals supporting research volume and field specificity, alongside general medical journals that amplify the reach and impact of key findings. Moreover, emerging interdisciplinary trends are evident through journals like International Journal of Molecular Sciences (m-index: 2.6) and Frontiers in Immunology (m-index: 2.714), which demonstrate rapid recent impact despite shorter publishing histories, while the inclusion of PLoS One (37 h-index, 2.056 m-index) reflects the growing influence of open-access platforms in disseminating psoriasis research to broader scientific audiences. A detailed overview of these bibliometric indicators is provided in Table 2, which summarizes the performance of the leading journals publishing on systemic psoriasis therapies.

**Figure 4:**
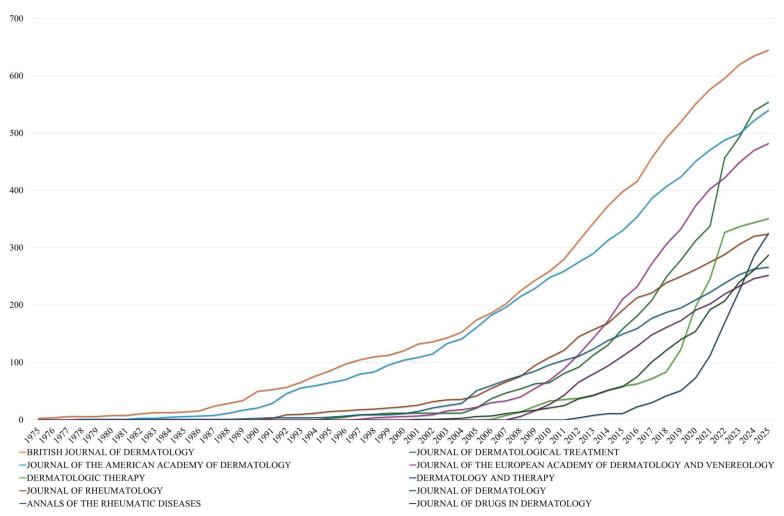
Annual cumulative publications in leading dermatology journals on systemic psoriasis therapies (1975–2025).

A study of the longitudinal publication patterns (Figure 4) among the leading dermatology journals reveals both steady expansion and distinct phases of acceleration. The British Journal of Dermatology (BJD) has demonstrated consistent publication since 1975, initially producing a mere three papers in its inaugural year and subsequently accumulating a total of 645 publications by 2025. It is noteworthy that its trajectory accelerated after 2000, with annual contributions showing a consistent increase, particularly between 2005 and 2020, during which output increased by almost threefold (from 174 to 551). This sustained growth is indicative of BJD’s long-standing role as a primary outlet for psoriasis-related dermatological research.

Conversely, the Journal of the American Academy of Dermatology (JAAD) exhibited a subsequent yet more pronounced escalation. While the journal’s initial output was negligible prior to 1980, it reached 540 cumulative publications by 2025, with its most rapid expansion occurring post-2005. The annual count increased from 161 in 2005 to 451 by 2020, representing an 180% increase. This indicates a heightened level of interest in systemic treatments within the field of clinical dermatology. A similar phenomenon is evident in The Journal of the European Academy of Dermatology and Venereology (JEADV), which, since its inception in 1997, has exhibited rapid and consistent growth, increasing from 1 article in 1997 to 482 by 2025. This trajectory, particularly the precipitous rise observed after 2010, is indicative of the journal’s growing influence within European dermatologic research networks.

Over the past twenty years, Dermatologic Therapy and The Journal of Dermatological Treatment have become increasingly important in the field. Dermatologic Therapy first appeared in the dataset in 2006, then rapidly grew to 351 publications by 2025, with a notable 78% increase occurring between 2020 and 2025 alone. The Journal of Dermatological Treatment has maintained a steady number of publications since 1995 and saw significant growth after 2015, rising from 181 to 554 articles in the subsequent ten years. Notably, the Journal of Drugs in Dermatology exhibited delayed but substantial growth, remaining absent from the dataset until 2008 before rapidly accumulating 252 publications by 2025, while Dermatology and Therapy demonstrated the most dramatic recent acceleration, emerging in 2012 and achieving 325 publications within just over a decade, representing a significant growth rate that underscores the field’s expanding therapeutic focus. These trends suggest a changing landscape in academic publishing, where specialized journals focusing on treatments are becoming more prominent alongside established titles. This shift toward specialized treatment-focused journals, evidenced by the parallel growth trajectories of Dermatology and Therapy alongside Journal of Dermatological Treatment, indicates both greater therapeutic diversity and an increased emphasis on translating research findings into practical clinical applications, reflecting the maturation of psoriasis pharmacotherapy from experimental interventions to established treatment protocols.

**Figure 5:**
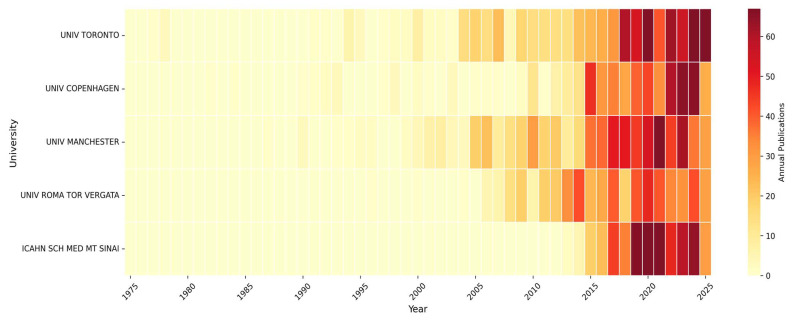
Heat map of institutional publication growth trajectories in systemic psoriasis research (1975–2025). Each cell represents publication counts per institution per year, with color intensity indicating publication volume. *X*-axis shows years at 5-year intervals, *Y*-axis lists top contributing academic institutions. Generated using Python with Seaborn and Matplotlib libraries.

The heatmap analysis of institutional publication output (Figure 5) reveals significant differences in research growth and momentum among leading centers in systemic psoriasis studies. The University of Toronto stands out as the top contributor, showing a long-term, steadily accelerating trajectory. Starting with a single publication in 1977, its output grew modestly until the mid-1990s, then expanded substantially, reaching 776 publications by 2025, with more than 60% of that growth occurring in the last five years. This suggests strong institutional investment and coordinated efforts across multiple research groups in dermatology and rheumatology. The University of Manchester and University of Copenhagen also exhibit strong, though distinct, growth paths: Manchester’s activity surged after 2000, doubling output between 2015 and 2025 to 728 publications, while Copenhagen grew more gradually since the 1980s with sharp momentum after 2010, increasing eightfold from 66 articles in 2014 to 538 by 2025.

Several newer institutions have shown rapid emergence in recent years. The Icahn School of Medicine at Mount Sinai began contributing in 2013, quickly accelerating from 2 to 536 publications by 2025, representing one of the fastest growth rates. Likewise, the University of Rome Tor Vergata expanded from minimal output before 2005 to 543 articles by 2025, reflecting strategic alignment with psoriasis research. These trends illustrate a maturing and evolving research landscape, where well-established institutions maintain dominance while newer and fast-growing centers increasingly shape global leadership. The heatmap highlights not just overall productivity, but also pivotal temporal shifts in institutional contributions to systemic psoriasis research.

The examination of the top 10 cited publications in systemic psoriasis therapy (Table 3) research reveals a transformative evolution spanning three decades (1991–2021), with collective citations totaling 14,630 and individual papers ranging from 1254 to 1709 citations. The temporal distribution demonstrates distinct research epochs: foundational nutritional-inflammatory investigations (Simopoulos et al., 1991 [27], Simopoulos et al., 2002 [28]) establishing omega-3 fatty acid deficiencies in Western diets, mechanistic TNF-α pathway elucidation (Bradley et al., 2008 [29]; Tracey et al., 2008 [30]), and the revolutionary shift toward IL-17/IL-23 axis targeting (Langley et al., 2014 [31]; Leonardi et al., 2008 [32]). Notably, the 2020–2021 period exhibited exceptional citation velocity, with Armstrong’s comprehensive review achieving 243.83 citations per year and Griffiths’ Lancet paper garnering 280.40 annual citations, indicating accelerated knowledge translation. The journal distribution across premier medical venues (NEJM, Lancet, JAMA representing 40% of publications) underscores psoriasis research’s elevation from specialized dermatology to mainstream medical priority. The prominence of safety-focused investigations among these highly cited works demonstrates the field’s commitment to comprehensive benefit-risk evaluation, with cardiovascular safety studies (Gelfand et al., 2006 [33], Neimann et al., 2006 [34]) and systematic safety assessments across therapeutic classes representing core research contributions that have fundamentally shaped clinical practice guidelines and regulatory approaches.

The therapeutic progression documented in these seminal works demonstrates quantifiable advances in treatment efficacy. Early nutritional interventions showed modest benefits when combined with conventional therapies, while TNF inhibitors established biological therapy’s viability. However, the paradigm shifts to IL-17A inhibition marked unprecedented therapeutic success, with Langley’s ERASURE/FIXTURE trials documenting 81.6% PASI 75 response rates for secukinumab 300 mg, significantly surpassing both placebo (4.5%, *p* < 0.001) and etanercept (44.0%, *p* < 0.001) [31]. Leonardi’s PHOENIX 1 trial similarly validated IL-12/23 targeting with ustekinumab achieving 67% PASI 75 response maintained over 76 weeks [32]. Critically, Gelfand’s 2006 identification of psoriasis as an independent cardiovascular risk factor (adjusted RR 3.10 for MI in severe psoriasis patients aged 30) fundamentally reconceptualized the disease from cutaneous to systemic inflammatory condition, influencing all subsequent therapeutic approaches [33]. This citation analysis reveals how psoriasis research has evolved from symptom management to comprehensive molecular targeting, with each paradigm maintaining relevance in contemporary integrated treatment strategies.

### 2.4. Scientific Mapping

**Figure 6:**
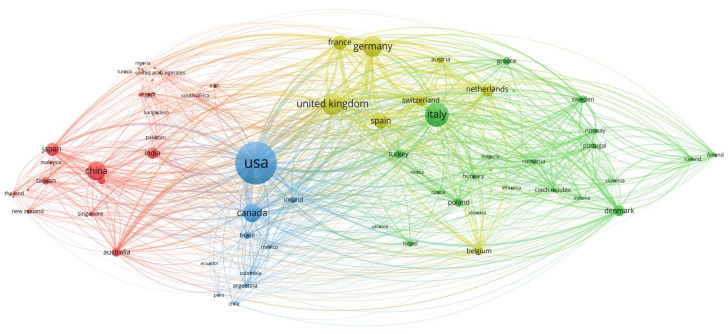
International collaboration clusters in psoriasis therapeutics research. Colors represent clusters of countries with stronger co-authorship links, as identified by network analysis.

The psoriasis therapeutics collaboration network (Figure 6) encompasses 70 countries organized into four distinct regional clusters, with the United States demonstrating the highest collaborative intensity (6455 TLS), followed by the United Kingdom (4564 TLS) and Germany (4236 TLS). The largest collaborative cluster (red cluster, 28 countries) forms a diverse Asia–Pacific–Africa–Middle East alliance anchored by China (698 TLS), Japan (1045 TLS), India (525 TLS), and South Korea (346 TLS), alongside significant Middle Eastern contributors including Saudi Arabia (155 TLS) and Egypt (163 TLS). The European research establishment (green cluster, 23 countries) centers on Italy (2509 TLS), supported by Denmark (1513 TLS), Sweden (1162 TLS), and Poland (667 TLS), while the Americas-focused cluster (blue cluster, 10 countries) demonstrates pronounced North–South dynamics with Canada achieving exceptional citation efficiency at 62.65 citations per document and substantial collaborative strength (3242 TLS), complemented by Latin American participation from Brazil (620 TLS), Mexico (368 TLS), and Argentina (485 TLS). The most compact yet influential cluster (yellow cluster, 8 countries) comprises Western European research leaders, with Germany, the United Kingdom, France (2571 TLS), and Switzerland (2251 TLS) forming an exceptionally dense network characterized by superior citation impacts, with Switzerland achieving 52.80 citations per document and Germany reaching 54.08 citations per document. Critical hub countries facilitate inter-cluster connectivity, with Australia (1196 TLS) bridging Asia-Pacific and Western research centers, while citation efficiency patterns reveal that smaller specialized economies like Estonia and Lithuania demonstrate that impactful psoriasis research transcends volume metrics, emphasizing collaborative quality in advancing therapeutic understanding.

**Figure 7:**
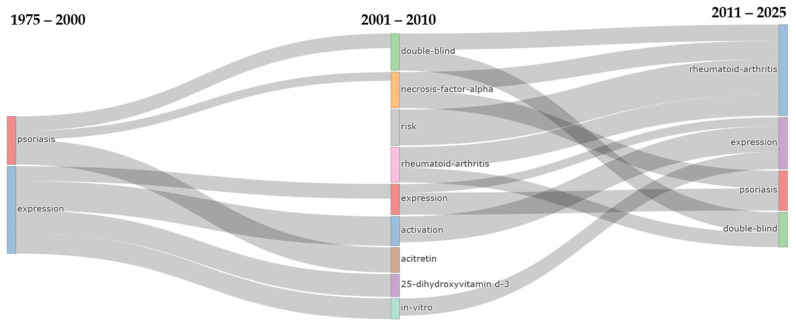
Sankey diagram illustrating thematic evolution in psoriasis therapeutics research across three dis-tinct periods (1975–2000, 2001–2010, 2011–2025). Flow thickness represents the strength of thematic connections between periods, with wider flows indicating stronger persistence or transformation of research themes. Generated using Bibliometrix 5.0.0package in R.

The thematic evolution analysis (Figure 7) across three distinct periods (1975–2000, 2001–2010, 2011–2025) reveals a systematic transformation from foundational disease research to sophisticated therapeutic methodologies and expanded disease scope. During the initial period, a focused approach was demonstrated with only two core themes: psoriasis and expression. These themes represented the foundational understanding of disease mechanisms and molecular processes. The intermediate period (2001–2010) witnessed dramatic thematic expansion to nine distinct research areas, with the original psoriasis theme generating substantial flows into TNF-α research (220 occurrences) and double-blind methodological approaches (168 occurrences). Meanwhile, the expression theme demonstrated remarkable persistence, maintaining continuity with 88 occurrences while simultaneously branching into specialized areas, including activation (50 occurrences), 25-dihydroxyvitamin D_3_ research (32 occurrences), and in vitro methodologies (25 occurrences). The emergence of rheumatoid arthritis as a significant theme during this period (90 occurrences from psoriasis) signaled the recognition of shared pathophysiological mechanisms between these inflammatory conditions. The contemporary period (2011–2025) demonstrated thematic consolidation into four primary domains, with rheumatoid-arthritis achieving the highest persistence strength (650 occurrences), followed by double-blind methodological approaches (571 occurrences), indicating the maturation of evidence-based research standards and the expansion of psoriasis therapeutics into broader autoimmune contexts. The expression theme demonstrated exceptional longevity across all periods, with sustained flows of 88 occurrences from 1975 to 2000 and 181 occurrences from 2001 to 2010. Substantial convergence occurred through TNF-α research, which contributed 283 occurrences to the psoriasis theme and 294 occurrences to the rheumatoid arthritis theme. This reflects the central role of TNF-α inhibition in contemporary systemic therapeutic approaches.

**Figure 8:**
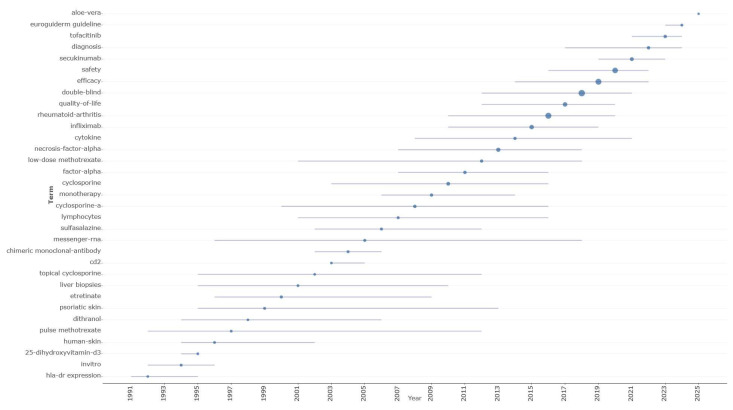
Chronological emergence of therapeutic terminology in psoriasis research. Each dot indicates the first occurrence year of a term in the literature, while the horizontal line represents its period of activity.

The way research terminology is distributed over time (Figure 8) shows clear phases of evolution in treatment, starting with basic mechanistic studies and moving on to conventional systemic treatments and finally arriving at contemporary targeted biological therapies. Early research (1992–1999) focused on understanding disease mechanisms to inform first-generation systemic treatments like methotrexate and cyclosporine and was dominated by HLA-DR expression (median year: 1992; number of documents: 9), in vitro studies (median year: 1994; number of documents: 39), 25-dihydroxyvitamin D3 research (median year: 1995; number of documents: 35), and basic human skin investigations (median year: 1996; number of documents: 32). This period also saw the development of early conventional treatments, including pulse methotrexate (median year: 1997; number of documents: 19) and dithranol (median year: 1998; number of documents: 15). The transitional period (2000–2009) demonstrated the emergence of systemic immuno-suppressive approaches, with etretinate achieving substantial research attention (2000, 126 documents), followed by cyclosporine-related studies including topical cyclosporine (2002, 22 documents) and cyclosporine-a (2008, 231 documents), while the first biological approaches appeared through chimeric monoclonal-antibody research (2004, 54 documents) and expanding messenger-RNA investigations (2005, 88 documents). The contemporary biological era (2010–2025) witnessed exponential growth in targeted therapy research, initiated by comprehensive cyclosporine studies (2010, 435 documents) and factor-α investigations (2011, 223 documents), culminating in the dominance of necrosis-factor-α research (2013, 750 documents) and specific TNF-α inhibitor studies led by infliximab (2015, 983 documents). The most recent period demonstrated the maturation toward evidence-based practice, with double-blind methodologies achieving peak research attention (2018, 3531 documents), efficacy assessments (2019, 2828 documents), and safety evaluations (2020, 2230 documents), while next-generation biologics emerged through secukinumab research (2021, 477 documents) and JAK inhibitor investigations including tofacitinib (2023, 217 documents), concluding with systematic guideline development evidenced by euroguiderm guideline terminology (2024, 43 documents).

**Figure 9:**
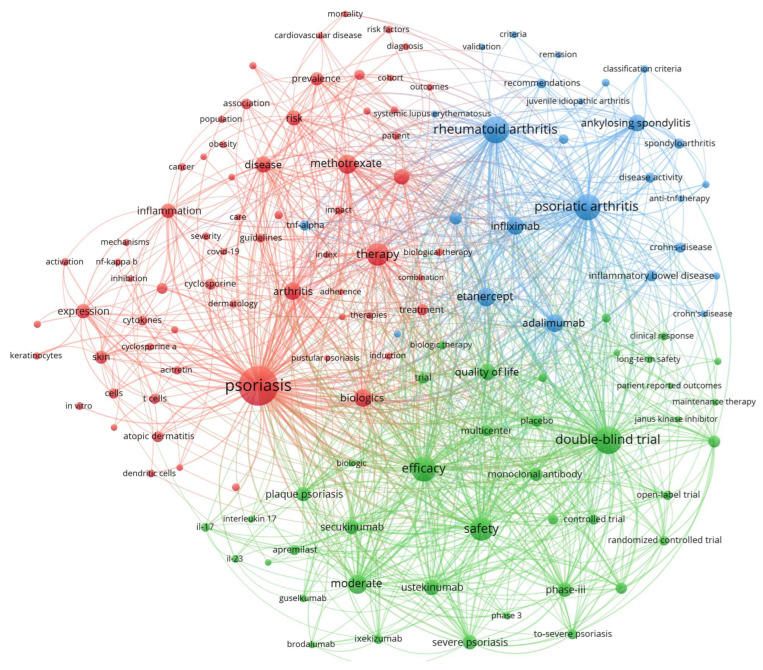
Thematic landscape of psoriasis therapeutics research revealed through keyword co-occurrence network analysis. Colors indicate clusters of keywords that frequently co-occur, reflecting distinct thematic areas within psoriasis pharmacotherapy research.

The keyword co-occurrence network analysis (Figure 9) revealed a highly interconnected research ecosystem comprising 132 nodes, demonstrating substantial thematic integration across psoriasis therapeutic domains. “Psoriasis” emerged as the dominant hub with 7283 occurrences and 25,621 total link strength, forming strongest connections with “safety” (1073 link strength), “adalimumab” (651 co-occurrences), and “psoriatic arthritis” (689 co-occurrences). The network architecture reveals safety research as integral to rigorous clinical methodology, with “safety” positioned prominently within the clinical trials cluster (green) alongside “double-blind trial”, “controlled trial”, and “phase-iii” studies. This network positioning demonstrates that safety evaluation represents core methodology rather than peripheral investigation, with strong connectivity to both therapeutic agents and evidence-based assessment frameworks. The network architecture reveals safety research as integral to rigorous clinical methodology, with “safety” positioned prominently within the clinical trials cluster (green) alongside “double-blind trial”, “controlled trial”, and “phase-iii” studies. This network positioning demonstrates that safety evaluation represents core methodology rather than peripheral investigation, with strong connectivity to both therapeutic agents and evidence-based assessment frameworks. The co-occurrence patterns indicate systematic integration of safety considerations across all major therapeutic classes, from conventional systemics through contemporary biologics.

The co-occurrence patterns indicate systematic integration of safety considerations across all major therapeutic classes, from conventional systemics through contemporary biologics. The network organized into three distinct thematic clusters: the red cluster focused psoriasis (65 items) anchored by “psoriasis”, the green cluster focused on clinical trials and therapeutics (41 items) centered on “double-blind trial” (3554 occurrences, 22,477 total link strength), and the blue cluster focused on inflammatory arthropathies (26 items) dominated by “rheumatoid arthritis” (3499 occurrences) and “psoriatic arthritis” (3325 occurrences). Contemporary IL-17/IL-23 inhibitors demonstrated recent research acceleration with guselkumab (316 occurrences, 2022.08 average publication year), ixekizumab (458 occurrences, 2020.92 average year), and secukinumab (1032 occurrences, 2020.77 average year) exhibiting strong integration with safety and efficacy terminology. The network’s high intra-cluster connectivity and systematic cross-cluster bridging through shared therapeutic targets illustrate psoriasis therapeutics research as a mature, methodologically rigorous field spanning conventional systemics through precision biologics with standardized evidence-based assessment frameworks.

## 3. Discussion

The global landscape of psoriasis therapeutics research reveals a multipolar architecture, with four regional clusters emerging to address the complex therapeutic challenges of this chronic inflammatory condition. The alliance of 28 countries in the Asia–Pacific, Africa, and the Middle East is a particularly significant development. China’s substantial collaborative strength, along with contributions from India, Japan, and Middle Eastern countries, indicates that psoriasis research has expanded far beyond traditional pharmaceutical centers. This geographic diversification likely reflects the recognition that psoriasis manifestations, treatment responses, and therapeutic needs vary significantly across different populations. This variation requires research approaches that capture diverse genetic backgrounds, environmental factors, and healthcare delivery contexts. The substantial research output from countries such as Saudi Arabia, Egypt, and South Korea indicates that psoriasis therapeutics research has evolved to embrace region-specific therapeutic development. This evolution acknowledges that effective treatments must account for population-specific disease characteristics and variations in healthcare infrastructure.

The striking contrast in publication volume and citation efficiency among the four clusters highlights the variety of research strategies and collaborative models in psoriasis therapeutics. Canada’s exceptional citation efficiency, at 62.65 citations per document, combined with its substantial collaborative network of 3242 TLS, exemplifies how strategic positioning within international research networks can amplify scientific impact beyond publication numbers alone. Meanwhile, the consistently high citation rates of the Western European cluster—Switzerland at 52.80 and Germany at 54.08 citations per document—demonstrate the continued importance of established pharmaceutical research infrastructure in producing influential psoriasis therapeutics studies. The network’s evolution toward inclusive, transcontinental collaboration patterns reflects the field’s maturation toward precision medicine approaches that recognize psoriasis as a heterogeneous condition requiring diverse therapeutic strategies tailored to specific patient populations, regulatory environments, and healthcare systems. This positions psoriasis therapeutics research as a model for global, collaborative approaches to complex dermatological conditions.

The thematic evolution in psoriasis therapeutics research demonstrates a sophisticated progression from disease-specific investigations to integrated autoimmune therapeutic frameworks, with the remarkable expansion from two foundational themes to nine distinct research domains during 2001–2010 reflecting the field’s response to breakthrough discoveries in cytokine biology and targeted therapy development. The substantial shift in research focus from psoriasis to TNF-α during this transformative period coincided with the clinical introduction of TNF-α inhibitors such as infliximab and etanercept. This shift fundamentally reshaped therapeutic paradigms, shifting the focus from conventional immunosuppression to targeted biological intervention. The persistence of expression themes across all three periods, with strengthening flows from 88 to 181 occurrences, underscores the continuous importance of molecular and genetic research in understanding psoriasis pathogenesis, while the emergence and consolidation of rheumatoid-arthritis as the dominant contemporary theme (650 occurrences) reflects the successful translation of psoriasis therapeutic advances to broader autoimmune conditions. This thematic convergence suggests that psoriasis research has evolved beyond dermatological boundaries, becoming a paradigm for autoimmune therapeutics with shared inflammatory pathways that enable cross-disease therapeutic applications.

The methodological evolution evidenced by the emergence and persistence of double-blind research approaches (571 occurrences in the contemporary period) illustrates the field’s maturation toward rigorous, evidence-based standards. Meanwhile, the consolidation of nine diverse themes into four core domains indicates the successful integration of mechanistic understanding with clinical application. The substantial bidirectional flows between rheumatoid-arthritis and double-blind themes (511 and 270 occurrences respectively) demonstrate how shared therapeutic targets have necessitated common methodological approaches across autoimmune conditions. This evolution positions psoriasis therapeutics research as a leader in precision medicine approaches to inflammatory diseases, where initial dermatological investigations have informed broader autoimmune therapeutic strategies. The thematic progression from foundational disease characterization to targeted therapeutic discovery and systematic evidence generation reflects the field’s successful translation of basic scientific discoveries into clinical innovations. This establishes psoriasis research as a model for translational medicine in chronic inflammatory conditions and paves the way for next-generation therapeutic approaches across the autoimmune disease spectrum.

The therapeutic keyword progression illustrates how psoriasis research has evolved from foundational mechanistic studies to sophisticated targeted intervention strategies over three decades. Early investigations centered on basic immunological processes, with hla-dr expression and 25-dihydroxyvitamin-d3 research establishing the inflammatory basis of disease pathogenesis, while conventional systemic treatments like etretinate (126 documents) and cyclosporine (435 documents) provided initial therapeutic advances despite significant limitations including hepatotoxicity and nephrotoxicity concerns. The revolutionary shift toward biological therapies emerged through targeted cytokine research, progressing from exploratory chimeric monoclonal-antibody studies to focused TNF-α investigations, culminating in landmark therapeutic breakthroughs with infliximab (983 documents) that fundamentally redefined treatment paradigms and patient outcomes expectations across inflammatory dermatological conditions.

Contemporary research priorities emphasize rigorous clinical validation methodologies, with unprecedented attention to double-blind study designs (3531 documents), systematic efficacy evaluations (2828 documents), and comprehensive safety assessments (2230 documents), reflecting regulatory demands for robust evidence supporting biological therapy approvals. The emergence of next-generation therapeutics, including IL-17 inhibitors like secukinumab and oral JAK inhibitors such as tofacitinib, demonstrates continued therapeutic innovation beyond TNF-α blockade, while systematic guideline development initiatives highlight successful knowledge translation into standardized clinical protocols. This progression exemplifies how dermatological research can drive broader therapeutic advances, with psoriasis-derived insights informing treatment strategies across multiple autoimmune conditions and establishing precedents for precision medicine implementation that considers genetic markers, disease severity indices, and individual treatment response profiles in therapeutic decision-making.

The evolution of psoriasis therapeutics research has unfolded through four distinct eras, each of which has fundamentally redefined our understanding of the disease and its treatment possibilities. The foundational era (1990s–early 2000s) established psoriasis as an immune-mediated condition and recognized the anti-inflammatory properties of omega-3 fatty acids. These fatty acids were found to have beneficial effects in patients with psoriasis when combined with conventional therapies setting the stage for targeted intervention strategies that would revolutionize treatment approaches. In individuals with psoriasis, the co-administration of eicosapentaenoic acid and docosahexaenoic acid alongside pharmacological treatments has been associated with improvements in cutaneous lesions, attenuation of etretinate-induced hyperlipidemia, and a reduction in cyclosporine-related toxicity [27].

The first biological revolution (2000–2008) introduced TNF-α inhibition as a breakthrough therapeutic strategy, with comprehensive understanding of TNF-mediated inflammatory pathways [29] enabling etanercept trials demonstrating 49% PASI 75 response rates at the highest dose compared to 4% with placebo [35], followed by infliximab achieving 80% PASI 75 responses [36], and adalimumab reaching 71% PASI 75 responses [37], fundamentally shifting treatment expectations from symptom control to substantial disease improvement. The field simultaneously evolved from treating localized skin disease to managing systemic inflammation and recognizing significant cardiovascular comorbidities. For example, 30-year-old patients with severe psoriasis have a 3.10-fold increased risk of myocardial infarction [33]. Comprehensive care guidelines for biological therapy management were also developed [38].

The precision targeting era (2008–2015) emerged from genetic discoveries that identified the IL-23/NF-κB pathways [39] and mechanistic insights that revealed distinct Th1 and Th17 T cell populations [40]. This led to the development of ustekinumab, which achieved 67.1% and 66.4% PASI 75 responses with 45 mg and 90 mg doses, respectively [32]. This was followed by the development of IL-17 antagonists, such as secukinumab, which achieved 81.6% and 77.1% PASI 75 responses in the ERASURE and FIXTURE trials [31], and ixekizumab reaching 71.4% PASI 90 responses with a 150 mg dose [41].

The contemporary optimization era (2015–present) has focused on selective IL-23 inhibition and oral targeted therapies, with guselkumab demonstrating superior efficacy compared to adalimumab with 85.1% versus 65.9% achieving clear/minimal disease ratings [42], while comprehensive UNCOVER phase 3 trials established ixekizumab’s superiority over etanercept [43], and JAK inhibitors emerged as oral alternatives [44]. It has been observed that anti-IL-17 and anti-IL-23 agents show sex-specific metabolic effects in psoriasis. IL-23 inhibition lowered visfatin in women and adiposity in men. Elevated baseline leptin correlated with reduced PASI-90 achievement, supporting its role as a biomarker for IL-23 inhibitor response prediction [45].

Biosimilars have emerged as important agents in psoriasis pharmacotherapy, providing highly effective and safe alternatives to reference biologics while maintaining comparable clinical outcomes. Adalimumab-fkjp, now FDA-designated as an interchangeable biosimilar to reference adalimumab, offers a new therapeutic option in moderate-to-severe plaque psoriasis, showing equivalent pharmacokinetics, efficacy, safety, and immunogenicity in switching and continuous treatment regimens [46]. Furthermore, SYSA1902, a biosimilar of ustekinumab targeting IL-12/23, demonstrated equivalent efficacy and safety to the reference drug in moderate-to-severe plaque psoriasis, achieving PASI 75 in 83.3% vs. 79.3% at week 12, offering a new therapeutic alternative in this patient population [47].

In a post hoc analysis of the EFFISAYIL 2 trial, spesolimab, an interleukin-36 receptor monoclonal antibody, achieved sustained improvement in skin symptoms in 63.3% and quality-of-life scores in 24.1% of generalized pustular psoriasis patients through 48 weeks, versus 29.0% and 3.2% with placebo [48].

In the Phase 3 open-label POETYK PSO-4 trial, deucravacitinib, an oral selective TYK2 inhibitor, demonstrated rapid and sustained PASI improvement through 52 weeks in Japanese patients with moderate-to-severe plaque psoriasis, including scalp and nail disease, with a favorable safety profile, representing an effective non-biologic targeted therapy option [49].

Topical therapies remain an integral component of psoriasis pharmacotherapy, with innovative formulations showing significant efficacy, safety, and skin barrier improvement, representing a novel non-immunosuppressive treatment option. A topical sponge containing Wharton Jelly-derived mesenchymal stem cell secretome enhanced with hyaluronic acid demonstrated safety and efficacy in psoriasis vulgaris, achieving up to 33% mPASI reduction, 41% plaque size decrease, and improved skin barrier function, representing a novel non-immunosuppressive therapeutic approach [50].

Recent trends in psoriasis pharmacotherapy emphasize the integration of drug repurposing strategies with nanotechnology and artificial intelligence. Repurposed agents such as curcumin, auranofin, niclosamide, and pentoxifylline have demonstrated anti-inflammatory and anti-psoriatic effects when formulated into advanced nanocarriers, liposomes, cerosomes, or nanostructured lipid carriers, improving cutaneous delivery, reducing systemic toxicity, and enhancing bioavailability. Preclinical models using imiquimod-induced psoriasiform inflammation confirmed that these nanoformulated compounds significantly reduce PASI scores, suppress pro-inflammatory cytokines, and attenuate histopathological skin changes. Concurrently, machine learning methods are being employed to accelerate drug screening by analyzing molecular descriptors, gene expression profiles, and drug-target interactions, facilitating the identification of novel candidates for topical or systemic repositioning. Integration of AI-driven design with nanocarrier-based drug delivery offers a transformative approach to psoriasis therapy, enhancing precision, minimizing systemic effects, and enabling scalable, clinically adaptable treatments [12].

This progression illustrates how sequential scientific breakthroughs, from identifying cytokines to elucidating genetic pathways to developing selective targeting strategies, have transformed psoriasis from a chronic condition with limited treatment options to a highly manageable disease with multiple therapeutic pathways that achieve unprecedented response rates. This has established psoriasis research as a paradigm for successful translational medicine that now informs therapeutic strategies development across numerous autoimmune and inflammatory conditions.

The comprehensive representation of safety research within our bibliometric analysis merits specific attention given its critical importance in psoriasis therapeutics. Analysis of our most highly cited publications reveals substantial coverage of long-term safety concerns, with cardiovascular risk assessment (i.e., Gelfand et al., 2006 [33], Neimann et al. 2006 [34]) and systematic safety surveillance (i.e., Roubille et al., 2015 [51]) among the most influential studies in the field. Dose–response relationships appear systematically across highly cited trials, including Leonardi’s etanercept dose-ranging study [35] and Papp’s comprehensive brodalumab dose–response evaluation across four concentration levels [52]. The keyword co-occurrence demonstrates that safety research is structurally embedded within rigorous clinical methodology frameworks, with “safety” co-occurring prominently alongside “double-blind trial”, “controlled trial”, and “phase-iii” studies, indicating systematic integration rather than isolated investigation. The temporal evolution from basic mechanistic safety studies through systematic adverse event monitoring to comprehensive surveillance protocols parallels regulatory requirements and therapeutic development phases. While highly specialized clinical pharmacology domains such as detailed pharmacokinetic studies may show different publication and indexing patterns, our analysis successfully captured the major safety research themes that have driven field advancement, including cardiovascular risk identification, malignancy surveillance, and therapeutic class-specific safety profiles that inform contemporary clinical practice.

The methodological approach demonstrated in this psoriasis pharmacotherapy analysis offers significant potential for investigating research evolution across diverse disease domains and therapeutic regimens. The framework’s adaptability is particularly valuable for chronic conditions with complex treatment landscapes, such as rheumatoid arthritis, inflammatory bowel disease, multiple sclerosis, and diabetes, where similar patterns of therapeutic evolution from conventional to targeted interventions may be observed. The AI-enhanced terminology normalization system addresses a universal challenge in medical bibliometrics, with the proliferation of synonymous terms and evolving nomenclature making it applicable in different situations. However, disease-specific considerations may require methodological adjustments, including search term weighting for rare diseases with limited literature, modified temporal periodization for rapidly evolving fields like gene therapy, and specialized validation protocols for emerging therapeutic modalities.

While this exhaustive bibliometric investigation offers valuable insights into the evolution of psoriasis therapeutics research, several methodological considerations merit acknowledgment. Although the Web of Science Core Collection ensures data uniformity and bibliometric reliability is essential for large-scale network analysis, our single-database approach represents a methodological trade-off between comprehensive coverage and analytical precision. This database selection prioritizes the consistent metadata structures, standardized citation linkages, and uniform quality control necessary for reliable co-occurrence mapping and temporal trend analysis across our 19,284-document dataset. The English-only inclusion criterion, though limiting multilingual research representation, was essential for consistent keyword analysis and terminology standardization using our AI-enhanced framework. Future investigations could explore hybrid approaches that balance broader linguistic inclusion with the analytical rigor requirements of bibliometric network construction.

Temporal analytical constraints introduce challenges to interpretation, particularly for publications from 2020 to 2025. These publications demonstrate artificially diminished citation metrics due to insufficient citation maturation periods. This temporal lag may inadvertently undervalue emerging therapeutic innovations, including novel JAK inhibitors, IL-23p19 inhibitors, and combination therapy approaches that represent current research frontiers. Despite attaining a high validation accuracy across pharmaceutical terminologies, the AI-enhanced terminology standardization system is incapable of comprehensively capturing the semantic intricacies and contextual nuances inherent in the evolving psoriasis therapeutic nomenclature, particularly about biosimilar designations and combination therapy protocols.

## 4. Materials and Methods

This bibliometric analysis addressing systemic therapies for psoriasis was conducted using the Web of Science Core Collection as the sole data source for literature acquisition. The decision to employ this database was based on methodological criteria aimed at ensuring analytical rigor and consistency. Web of Science offers detailed and structured bibliographic entries, enriched with extensive metadata such as full author affiliations, citation linkages, and keyword indexing schemes, which support the implementation of advanced bibliometric visualization and mapping tools. Additionally, the database’s stringent peer-review policies and curated indexing practices contribute to the inclusion of scientifically robust publications. Its standardized data formats and uniform field structures reduce processing difficulties and enhance the accuracy of inter-record linkages and comparative assessments. Relying on a single, authoritative database avoided the integration challenges often associated with using multiple sources, thereby improving both the reproducibility and methodological validity of the bibliometric framework employed in analyzing the literature on psoriasis treatments [53,54]. The chosen analytical framework (i.e., VOSviewer 1.6.20, Bibliometrix 5.0.0) requires uniform data structures for keyword mapping, temporal analysis, and thematic evolution tracking. Web of Science’s standardized field coding ensures consistent processing across the 19,284-document dataset, while mixed-source approaches introduce systematic biases through varying metadata completeness and indexing protocols.

This investigation employs bibliometric analysis methodology, which examines research patterns through computational analysis of publication metadata, citation networks, and keyword structures rather than full-text content evaluation. Unlike systematic reviews that require individual article assessment, bibliometric studies analyze aggregate data patterns including publication trends, author collaborations, institutional affiliations, citation metrics, and thematic evolution using specialized computational tools. This methodology enables large-scale pattern recognition across extensive datasets that would be impractical for manual review approaches.

**Figure 10:**
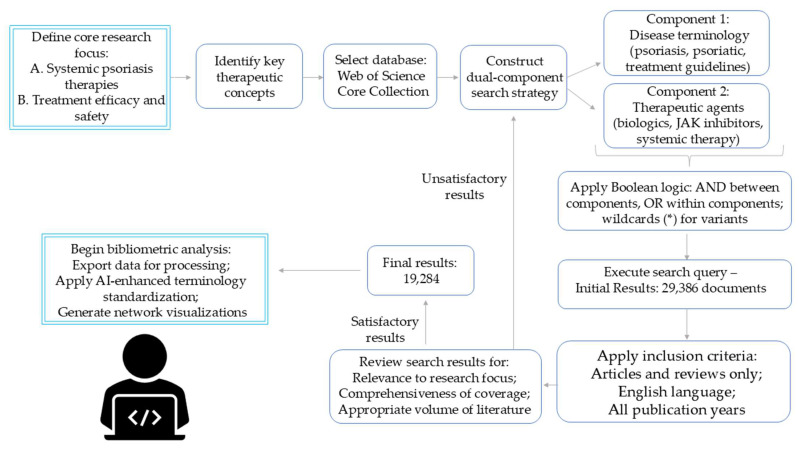
Methodological flowchart depicting the systematic literature search and bibliometric analysis workflow. The diagram illustrates the progression from research focus definition through dual-component search strategy construction, database querying (Web of Science Core Collection), inclusion criteria application (29,386 to 19,284 documents), and final AI-enhanced bibliometric analysis with network visualization generation. The asterisk * denotes the use of wildcard characters in the search query to capture multiple word variants.

The literature search employed a two-component strategy targeting publications on psoriasis and its systemic treatments. The first component captured disease-related terminology: “psoriasis”, “psoriatic”, “psoriasis pharmacotherapy”, “psoriasis treatment”, and “treatment guideline*”. The second component encompassed therapeutic agents, including conventional systemic drugs (methotrexate, cyclosporine, acitretin, apremilast), JAK inhibitors (tofacitinib, baricitinib), and various biologics targeting different pathways. Additional search terms included broader categories: “JAK inhibitor*”, “biologic*”, “systemic therapy”, “targeted therapy”, “IL-23 inhibitor*”, and “TNF inhibitor*”. These components were connected using AND logic operator to identify publications discussing psoriasis treatments specifically. Alternative terms within each component were linked with OR operators to maximize retrieval. Wildcard symbols (*) captured variant word endings, ensuring comprehensive coverage of relevant literature while maintaining search focus on systemic psoriasis therapeutics. A systematic filtering process was then applied to refine this dataset for analysis (Figure 10).

The database search identified 29,386 documents covering all available publication years in Web of Science. Selection criteria were limited to articles and reviews published in English. This approach ensured consistency for text-based analyses, including keyword mapping and content interpretation. After applying these filters, the dataset comprised 19,284 documents, reduction from the initial results. This refinement process maintained comprehensive coverage of psoriasis treatment research while excluding publication types that typically contribute minimally to citation networks and bibliometric indicators. The resulting dataset provided a linguistically uniform collection suitable for co-occurrence analysis and thematic exploration of systemic psoriasis therapies.

The analytical framework integrated several complementary software environments to examine collaborative structures and temporal research patterns within systemic psoriasis therapy literature. VOSviewer (version 1.6.20) [55] functioned as the core visualization platform, enabling the construction of network maps that illustrated keyword association patterns and international research partnerships. Temporal trend examination across psoriasis therapeutic research was performed through the Bibliometrix package (version 5.0.0) [56], accessed via the Biblioshiny interface within the R computational environment [57]. Microsoft Excel facilitated initial data processing, statistical summarization, and generation of supporting graphical representations, enhancing the accessibility and comprehension of complex bibliometric findings.

Different software tools count publications in different ways, which can lead to varying results. One tool splits the credit when multiple countries work together on a research paper—each country receives a portion based on their contribution. The other tool gives full credit to every country involved in the collaboration. These different counting methods become more noticeable for countries that have increased their international partnerships in recent years. The splitting method gives a more careful estimate of what each country contributed on their own, while the full-credit method shows the complete picture of how much each country participates in international research.

Publication patterns over time for top-contributing countries were displayed using Python 3.12.3 [58] along with several data analysis tools: Pandas version 2.3.1 [59], Matplotlib version 3.10.3 [60], Seaborn version 0.13.2 [61], and NumPy version 2.3.1 [62]. The data for this analysis was exported from the Bibliometrix interface. The total publication numbers were broken down into yearly amounts and arranged in tables showing each country’s output per year. Visual heat maps were built to show how active each country was in research, with grid lines to make the charts easier to read and labels every 5 years along the bottom. This method helped us spot research trends over time and see how different countries’ research output changed during the 51-year study period. All bibliometric indicators (e.g., h-index, g-index, m-index etc.) are calculated specifically for psoriasis-related publications within each journal, enabling comparison of journal performance within this therapeutic domain. This domain-specific approach allows for meaningful comparison of how different journals perform in publishing impactful psoriasis therapeutics research, which would not be possible using general journal metrics that include diverse medical topics.

The study of collaboration networks between countries included only nations that published at least 10 documents to ensure reliable partnership patterns, while leaving out countries with too few publications for accurate network analysis. In the network charts, the size of each circle showed how many total publications each country had, with bigger circles representing countries that produced more research. The thickness of the lines connecting countries showed how strong their research partnerships were, with thicker lines showing more frequent and closer collaboration between nations. To make sure the data was accurate, we manually corrected different name variations in order to reduce biases (for example, United Kingdom and Wales were standardized under one name).

The Bibliometrix package (version 5.0.0) and its specialized tools were used for tracking long-term research patterns. We examined how research topics evolved by creating flow diagrams that showed how themes changed across three carefully chosen time periods: 1975–2000, 2001–2010, and 2011–2025. These periods were selected to match major developments in psoriasis treatment—the early foundational years (1975–2000), the revolutionary biological therapy introduction period (2001–2010), and the modern era of diversified targeted treatments (2011–2025). Only themes that appeared in at least 100 documents per period were included to ensure the results were statistically meaningful. We also analyzed when specific research terms first appeared and how long they stayed important in the field, which helped us understand how research priorities shifted over the five decades.

A specialized AI-driven framework was developed for identifying synonymous terms and standardizing terminology within psoriasis therapeutic literature. This computational system utilizes three integrated approaches: exact text pattern matching through regular expressions to detect formatting variations, semantic clustering using transformer models (BioBERT and SentenceTransformers) [63,64] with DBSCAN clustering (cosine similarity threshold ≥ 0.90) [65], and context-aware fuzzy matching (FuzzyWuzzy library) [66] that recognizes psoriasis-specific therapeutic components including biologics, systemic treatments, and intervention strategies.

The framework incorporates validation rules for dermatological terminology, manages computational dependencies through fallback mechanisms, and employs graph-based merging (NetworkX) to consolidate overlapping synonym groups while maintaining accuracy through minimum group size thresholds. Input terminology undergoes preprocessing to standardize formatting and extract core therapeutic elements, with outputs generated as comma-separated files compatible with VOSviewer software for bibliometric mapping applications.

**Figure 11:**
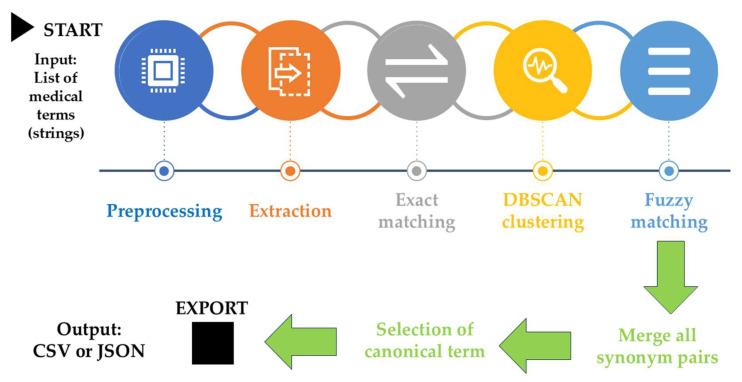
The system architecture underlying the pipeline for medical term normalization and synonym resolution. Initially, a list of input terms is subjected to preprocessing and vector representation. A sequential matching framework is then applied, combining exact string comparison, semantic similarity, and fuzzy logic techniques. Identified synonym pairs are integrated into a graph-based structure, from which canonical terms are determined for each connected component. The resulting output is a structured dictionary that links each canonical term to its associated lexical variants.

System accuracy was evaluated using a stratified random sample of 300 term pairs from the generated synonym collection. Three psoriasis research specialists independently assessed each synonym group for semantic accuracy based on equivalence criteria. The system was considered validated when all three evaluators achieved unanimous agreement on ≥90% of the terminological groupings. The AI-enhanced terminology standardization specifically addressed the challenge of semantic variation in pharmaceutical nomenclature within bibliometric datasets. This computational approach was designed to process extracted keywords and abstracts for terminology consistency, not to replace human content evaluation. The configuration of the system is visually represented in Figure 11.

## 5. Conclusions and Prospects

The analysis shows how psoriasis studies have completely changed our understanding of autoimmune disease treatment and created new approaches for personalized medicine. The field shows remarkable growth, with annual publications increasing from 9 articles in 1975 to 1638 in 2024. This progression includes four distinct developmental phases: exploratory foundations (1975–1990), mechanistic understanding (1991–2004), biological therapy revolution (2005–2015), and precision targeting era (2016–2025). The most dramatic acceleration occurred during 2005–2015, when research output surged from 262 to 742 annual publications, coinciding with the widespread adoption of TNF-α inhibitors for psoriasis treatment that revolutionized therapeutic approaches.

The documented research trajectory suggests several promising therapeutic avenues emerging from identifiable bibliometric patterns. Our keyword co-occurrence analysis reveals recent research acceleration in IL-17/IL-23 inhibitor studies, with guselkumab, ixekizumab, and secukinumab showing strong contemporary research momentum, indicating continued innovation within this therapeutic class. The chronological emergence of JAK inhibitor terminology, particularly tofacitinib research documented through 2023, combined with systematic guideline development initiatives, suggests that oral targeted therapies will likely experience continued research expansion and clinical optimization. The thematic evolution analysis demonstrates increasing convergence between psoriasis and rheumatoid arthritis research domains, indicating that future therapeutic development will likely leverage shared inflammatory pathway insights and cross-disease treatment applications. Additionally, the consistent emphasis on evidence-based methodologies and safety integration across all therapeutic classes in our network analysis suggests that future research will prioritize comprehensive risk–benefit evaluation and systematic comparative effectiveness studies to optimize treatment selection and personalization.

The study of international collaboration shows that 70 countries work together in four main regional groups. While the United States produces the most research with 5959 publications, other countries achieve higher impact per paper, with Canada reaching 62.65 citations per document and the Netherlands achieving 60.88. The formation of a large 28-country group spanning Asia, the Pacific, Africa, and the Middle East shows a major shift toward including more regions in psoriasis research to address the disease’s global impact and different population needs.

The multipolar research landscape identified in our collaboration network analysis demonstrates a fundamental shift from Western-dominated research to globally distributed innovation, with the emergence of a 28-country Asia–Pacific–Africa–Middle East alliance representing the largest collaborative cluster. China’s dramatic research expansion, evidenced by its growth from minimal publications to substantial output, illustrates the rapid emergence of new research centers that could alter the traditional geographic distribution of therapeutic innovation. The thematic evolution analysis shows consolidation from nine diverse research domains into four integrated areas focused on targeted therapeutics and evidence-based methodologies, indicating increased research coordination and systematic approaches to therapeutic development. The persistent strength of international collaboration networks, with 70 countries participating across four regional clusters, demonstrates that future therapeutic research will likely continue to benefit from diverse geographic perspectives and shared research expertise.

Thematic evolution demonstrates intellectual maturation through systematic progression from two foundational themes (1975–2000) to nine specialized domains (2001–2010), ultimately consolidating into four integrated areas focused on targeted therapeutics and evidence-based methodologies (2011–2025). This reflects successful translation of immunological discoveries into clinical innovations, with sustained emphasis on rigorous research standards. The therapeutic terminology timeline illustrates three innovation waves: foundational immunological research (1992–1999), conventional systemic treatments (2000–2009), and contemporary biological targeting (2010–2025). This evolution progressed from basic mechanistic studies through TNF-α inhibition breakthroughs to current IL-17/IL-23 pathway selectivity and emerging JAK inhibitor development, transforming treatment expectations from symptom control to substantial disease remission.

Our bibliometric analysis demonstrates that psoriasis research has established connections with broader autoimmune research domains, as evidenced by the substantial thematic convergence with rheumatoid arthritis research documented in our evolution analysis. The systematic consolidation from nine diverse research themes into four integrated areas focused on targeted therapeutics and evidence-based methodologies reflects increasing research coordination and systematic approaches to therapeutic evaluation. The successful application of our AI-enhanced terminology normalization framework to a 19,284-document dataset demonstrates the potential for large-scale bibliometric analysis to map therapeutic research evolution patterns that could inform strategic research planning across medical disciplines.

These findings demonstrate that psoriasis treatment research serves as a prime example of successful translational medicine, where basic research discoveries have led to major treatment breakthroughs with wide applications in other autoimmune diseases. As personalized medicine continues to develop, this analysis provides important insights for researchers, pharmaceutical companies, and healthcare systems working to understand the changing treatment field and develop future therapeutic approaches.

## Figures and Tables

**Table 1:** Publication output, citation metrics, and collaboration strength for the top 10 most productive countries in systemic psoriasis therapy research.

Country	Documents	Citations	Average Citations/Doc.	TLS
USA	5959	300,339	50.40	6455
Italy	2356	636,75	27.03	2509
United Kingdom	2021	103,530	51.23	4564
Germany	1819	98,379	54.08	4236
China	1598	27,530	17.23	698
Canada	1491	93,416	62.65	3242
Spain	1105	42,553	38.51	2338
France	1057	57,022	53.95	2571
Japan	881	31,984	36.30	1045
The Netherlands	874	53,211	60.88	2286

TLS, total link strength.

**Table 2:** Leading journals in psoriasis therapies, citation metrics and publication output.

Source	h_Index	g_Index	m_Index	Total Citations	Publications No.	Publication Start Year
Journal of the American Academy of Dermatology	105	173	2.283	41,319	540	1980
Annals of the Rheumatic Diseases	95	158	1.979	28,451	266	1978
British Journal of Dermatology	93	141	1.824	35,348	645	1975
Journal of Investigative Dermatology	69	113	1.353	15,331	233	1975
Journal of the European Academy of Dermatology and Venereology	65	99	2.241	17,686	482	1997
Journal of Rheumatology	59	83	1.686	11,275	324	1991
Arthritis and Rheumatism	56	72	1.333	12,592	72	1984
Rheumatology	55	85	2.037	9418	247	1999
Archives of Dermatology	53	92	1.039	9057	120	1975
Lancet	50	53	1.064	20,250	53	1979
Arthritis Research & Therapy	45	75	1.875	6470	150	2002
Dermatology	42	55	1.273	5193	213	1993
American Journal of Clinical Dermatology	41	60	1.783	4903	161	2003
Jama Dermatology	41	69	3.154	4963	101	2013
Journal Of Dermatological Treatment	41	59	1.323	8731	554	1995
Journal Of Dermatology	40	59	1.6	5735	287	2001
International Journal of Molecular Sciences	39	70	2.6	5559	165	2011
Frontiers In Immunology	38	64	2.714	4999	196	2012
Acta Dermato-Venereologica	37	55	0.725	4692	203	1975
Plos One	37	62	2.056	4291	111	2008

h-index, Hirsch index; g-index, Egghe’s g-index; m-index, m-quotient. Citation metrics represent performance of psoriasis-related articles only, not entire journal metrics.

**Table 3:** Top 10 most-cited publications in systemic psoriasis therapy research (1991–2021).

Author/Year/Journal	TC	TC/Year	Normalized TC	DOI
Simopoulos Ap, 1991, Am J Clin Nutr	1709	48.83	20.46	10.1093/ajcn/54.3.438
Langley RG, 2014, N Engl J Med	1613	134.42	36.20	10.1056/NEJMoa1314258
Bradley Jr, 2008, J Pathol	1554	86.33	20.73	10.1002/path.2287
Gelfand JM, 2006, Jama-J Am Med Assoc	1491	74.55	22.65	10.1001/jama.296.14.1735
Armstrong AW, 2020, Jama-J Am Med Assoc	1463	243.83	52.94	10.1001/jama.2020.4006
Simopoulos AP, 2002, J Am Coll Nutr	1454	60.58	19.53	10.1080/07315724.2002.10719248
Griffiths CEM, 2021, Lancet	1402	280.40	69.55	10.1016/S0140-6736(20)32549-6
Singh JA, 2016, Arthritis Rheumatol	1351	135.10	33.64	10.1002/art.39480
Leonardi CL, 2008, Lancet	1339	74.39	17.86	10.1016/S0140-6736(08)60725-4
Tracey D, 2008, Pharmacol Ther	1254	69.67	16.73	10.1016/j.pharmthera.2007.10.001

TC, total citations.

## Data Availability

The raw data supporting the conclusions of this article will be made available by the authors on request.

## Abbreviations

The following abbreviations are used in this manuscript:

TNF-α	Tumor Necrosis Factor alpha
IL-12/17/23	Interleukin 12/17/23
JAK	Janus Kinase
PASI	Psoriasis Area and Severity Index
TLS	Total Link Strength
TC	Total Citations
TYK2	Tyrosine Kinase 2
h-index	Hirsch index
g-index	Egghe’s g-index
m-index	m-quotient (h-index/time)
